# *Mangrovivirga cuniculi* gen. nov., sp. nov., a moderately halophilic bacterium isolated from bioturbated Red Sea mangrove sediment, and proposal of the novel family *Mangrovivirgaceae* fam. nov.

**DOI:** 10.1099/ijsem.0.004866

**Published:** 2021-07-02

**Authors:** Fatmah O. Sefrji, Grégoire Michoud, Ramona Marasco, Giuseppe Merlino, Daniele Daffonchio

**Affiliations:** ^1^​ Biological and Environmental Sciences and Engineering Division (BESE), Red Sea Research Center (RSRC), King Abdullah University of Science and Technology (KAUST), Thuwal, Saudi Arabia

**Keywords:** *Bacteroidetes*, cultivation, mangrove crabs, *Mangrovivirgaceae*, *Mangrovivirga*, mangrove sediments

## Abstract

A strictly aerobic, Gram-stain-negative, non-motile, rod-shaped bacterium, designated strain R1DC9^T^, was isolated from sediments of a mangrove stand on the Red Sea coast of Saudi Arabia via diffusion chamber cultivation. Strain R1DC9^T^ grew at 20–40 °C (optimum, 37 °C), pH 6–10 (optimum, pH 8) and 3–11 % NaCl (optimum, 7–9 %) in the cultivation medium. The genome of R1DC9^T^ was 4 661 901 bp long and featured a G+C content of 63.1 mol%. Phylogenetic analyses based on the 16S rRNA gene sequence and whole-genome multilocus sequence analysis using 120 concatenated single-copy genes revealed that R1DC9^T^ represents a distinct lineage in the order *Cytophagales* and the phylum *Bacteroidetes* separated from the *Roseivirgaceae* and *Marivirgaceae* families. R1DC9^T^ displayed 90 and 89 % 16S rRNA gene sequence identities with *Marivirga sericea* DSM 4125^T^ and *Roseivirga ehrenbergii* KMM 6017^T^, respectively. The predominant quinone was MK7. The polar lipids were phosphatidylethanolamine, two unknown phospholipids and two unknown lipids. The predominant cellular fatty acids were the saturated branch chain fatty acids iso-C_15 : 0_, iso-C_17 : 0_ 3-OH and iso-C_17 : 0_, along with a low percentage of the monounsaturated fatty acid C_16 : 1_ *ω*5*c*. Based on differences in phenotypic, physiological and biochemical characteristics from known relatives, and the results of phylogenetic analyses, R1DC9^T^ (=KCTC 72349^T^=JCM 33609^T^=NCCB 100698^T^) is proposed to represent a novel species in a new genus, and the name *Mangrovivirga cuniculi* gen. nov., sp. nov. is proposed. The distinct phylogenetic lineage among the families in the order *Cytophagales* indicates that R1DC9^T^ represents a new family for which the name *Mangrovivirgaceae* fam. nov. is proposed.

Mangroves represent one of the most productive ecosystems, playing an important role in nutrient cycling and energy flow at the dynamic interface between land and sea [1, 2]. Mangrove sediments harbour an enormous microbial biomass (1×10^9^–1×10^11^ cells per gram of sediment [3, 4]) rich in functional and phylogenetic diversity [5–7]. *Bacteroidetes* account for a significant fraction of the bacterial community (*i.e.*, 5–45 % in mangrove sediments and contiguous environmental niches such as mangrove plant tissues [6–10]), indicating their importance in marine ecosystems, similar to other coastal and offshore sediments, seawater and hydrothermal vents [11–14].

The phylum *Bacteroidetes* comprises four classes: *Bacteroidia*, *Flavobacteriia*, *Sphingobacteriia* and *Cytophagia* [15]. The taxonomy of this last class—including only the order *Cytophagales*—was recently revised with the inclusion of the families *Cesiribacteraceae*, *Flexibacteraceae*, *Fulvivirgaceae*, *Marivirgaceae*, *Reichenbachiellaceae*, *Roseivirgaceae* and *Thermoflexibacteraceae* [16–21]. Notably, the number of recognized genera in the phylum *Bacteroidetes* has increased exponentially [22–25], suggesting that this phylum contains untapped diversity.

In this study, we describe *Mangrovivirga cuniculi* gen. nov., sp. nov. (R1DC9^T^), which was isolated from crab-bioturbated mangrove sediment of the Red Sea coast, as the first representative species of a new genus (*Mangrovivirga*) and family (*Mangrovivirgaceae*) within the order *Cytophagales*.

## Isolation and habitat

**Fig. 1.**
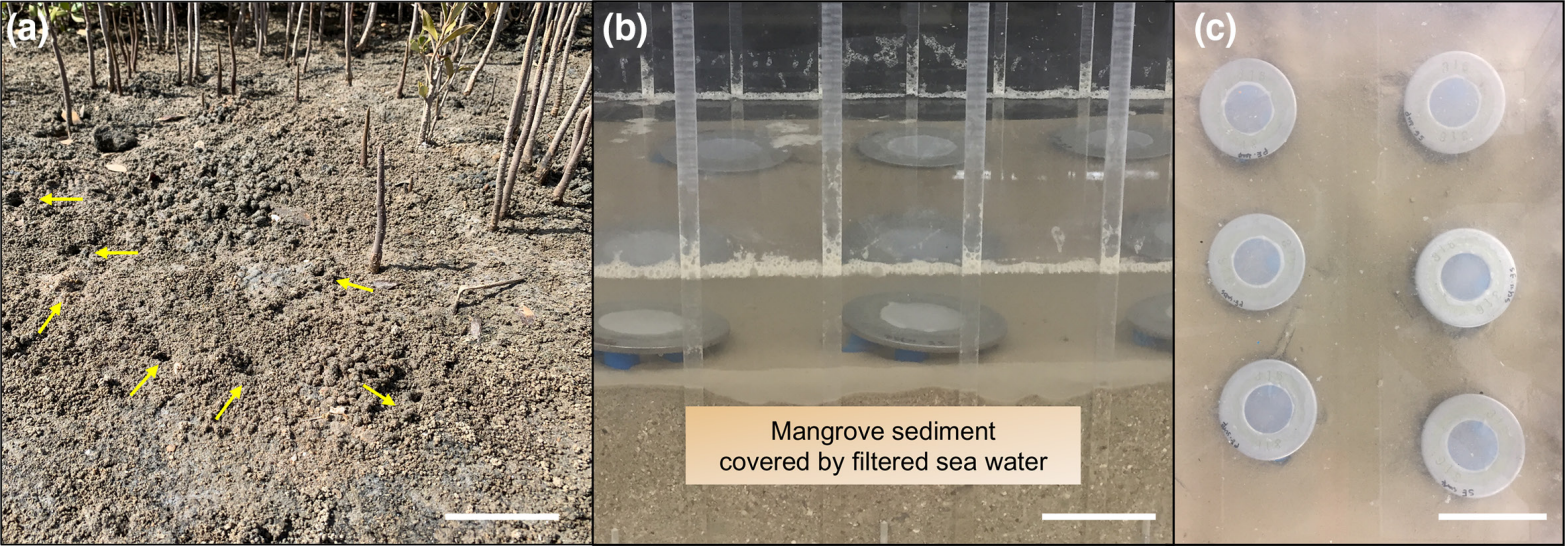
(**a**) Sediments in the mangrove forest at KAUST bioturbated by *Uca* species crabs; crab barrows are indicated by yellow arrows. Leaves forming mangrove litter are also visible. Bar, 10 cm. (**b**) Aquarium filled with mangrove bioturbated sediments and FSW for the incubation of DCs; bar, 6 cm. (**c**) Aerial view of DCs placed on the surface of mangrove sediments and cover with FSW inside the aquarium; bar, 6 cm.

Strain R1DC9^T^ was isolated from mangrove sediment bioturbated by crabs of the genus *Uca* at the Ibn Sina Field Research Station and Nature Conservation Area (22.34° N, 39.09° E), a coastal area encompassing 152 hectares at the King Abdullah University of Science and Technology (KAUST). For isolation, a diffusion chamber (DC)-based approach [26] was used. Crab-bioturbated mangrove sediments (Fig. 1a) were sampled with sterile spoons and used as inocula for bacteria cultivation experiments. Aliquots of sediment and dead mangrove leaves from the site were also sampled for use as supplementary nutrient sources in the growth medium. Briefly, the DC contained an agar seawater matrix [1.5 % agar in filtered seawater (FSW) from the Red Sea] supplemented with 0.1 % sediment extract inside a confined environment (stainless steel washer) that was separated from the environment by two 0.03 µm pore-size polycarbonate membranes. Sediment extracts were prepared by mixing sediments with Milli-Q water at a 1 : 10 ratio. The obtained mixture was autoclaved for 30 min, centrifuged for 10 min at 11 000 r.p.m. and then filter-sterilized using 0.22 µm pore-size filters. The melted DC agar matrix (40 °C) was inoculated with bioturbated sediment diluted in FSW to 1×10^−4^ [27]. The sealed DCs were incubated in an aquarium (30×46×30 cm^3^) filled with the original sediments (5–8 cm thick layer) and seawater (5 cm thick layer above the sediments; Fig. 1b, c) for 21 days at room temperature (25±2 °C). After incubation, the DCs were opened under sterile conditions in a flow hood, and the FSW agar was homogenized via passage through a syringe equipped with a 25-gauge needle, diluted with FSW, and mixed with medium (molten FSW agar 1.5 % w/v and sediment extract at a final concentration of 0.1 % v/v) to a final dilution of 1×10^−4^. The obtained microbial culture was used to prepare standard plate cultures on Petri dishes containing FSW agar supplemented with 0.1 % sediment extract (incubated at 25 °C for 14 days). Colonies that grew on the dishes were further collected and picked up using long-tip glass Pasteur pipettes (Sigma-Aldrich), followed by sub-culture on plates containing 1 l 0.1×lysogeny broth (LB; supplemented with 10 g of tryptone, 5 g yeast extract and 20 g agar) prepared with FSW and supplemented with 0.1 % sterile sediments. R1DC9^T^ inoculated on this medium was grown under aerobic conditions at 25 °C for 4 days to ensure bacterial colony development. This last step was repeated three times to obtain pure bacterial colonies. Pure cultures of R1DC9^T^ were stored in 30 % glycerol at −80 °C and routinely cultivated in 1×LB prepared with FSW (therefore, LB+FSW). This medium had a final salinity of 4 % based on refractometry.

## Phylogeny based on the 16S rRNA and genome sequences

Fresh bacterial cultures grown in LB+FSW at 37 °C were used for total genomic DNA extraction using the Maxwell RSC Automated Nucleic Acid Purification system and a Maxwell RSC Cultured Cells DNA kit (Promega). The DNA concentration was quantified using a Qubit dsDNA assay and a high-sensitivity kit (Thermo Fisher Scientific). DNA quality was examined via electrophoresis on 1 % agarose gels with Bioanalyzer 2100 (Agilent). The genome of R1DC9^T^ was sequenced using a PacBio RS2 sequencer (Pacific Biosciences) at the Bioscience Core Lab (KAUST, Saudi Arabia). The reads were assembled using SMRT analysis software (PacBio) and the HGAP.3 workflow [28]. The genome was annotated using the automated annotation pipeline Prokka [29]. The annotation and functionality of the new species were completed using RAST and KEGG [30–32]. The 16S rRNA gene sequence obtained from the R1DC9^T^ genome was analysed using the RDP Classifier and blast to search the NCBI database for all available 16S rRNA sequences. Phylogenetic trees were reconstructed using the maximum-likelihood and neighbour-joining methods available in the megaX software package [33]. The topologies of the phylogenetic trees were evaluated via bootstrap analyses (based on 1000 replicates). Multilocus sequence analysis (MLSA) of the phylogenetic tree was performed with 120 concatenated single-copy genes using the GTDB-Tk software [34]. A bootstrap analysis of 1000 replicates was used to evaluate the tree topology [35]. *In silico* DNA–DNA hybridization (DDH), blast-based average nucleotide identity (ANIb), and average amino acid identity (AAI) scores of R1DC9^T^ and related species were calculated using GGDC, JSpeciesWS and AAI-profiler, respectively [36–39], with the default parameters. The 16S rRNA gene and whole-genome sequences were deposited in GenBank under the accession numbers MT146883 and CP028923.1, respectively.

**Fig. 2.**
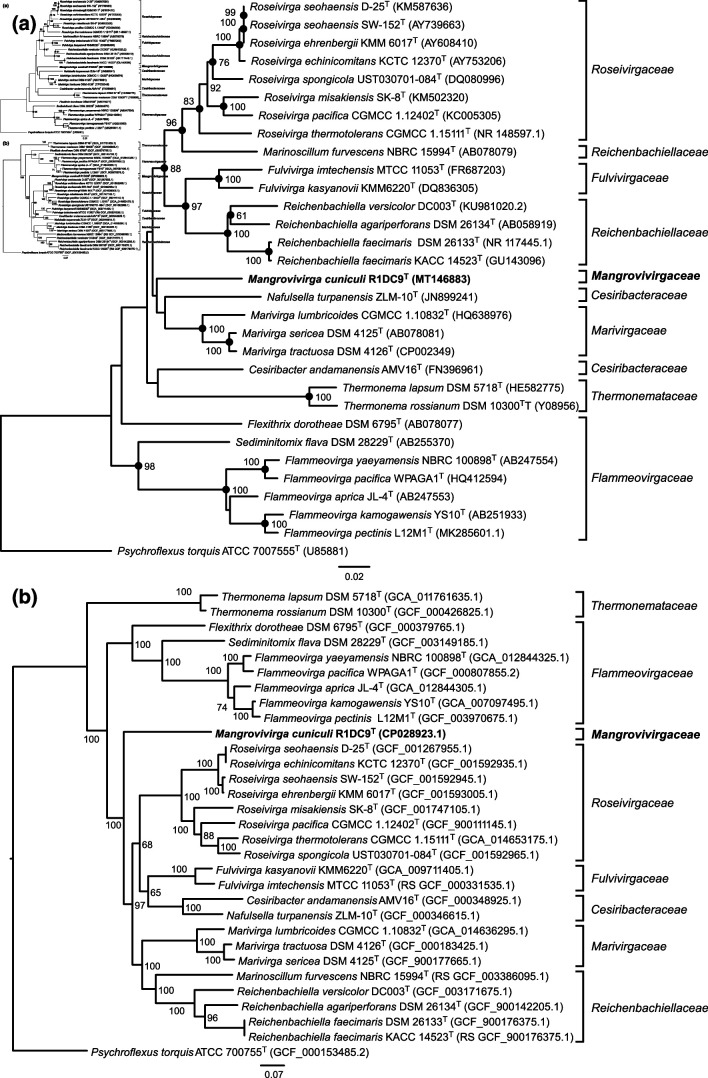
(**a**) Maximum-likelihood phylogenetic tree based on the 16S rRNA gene sequences presenting the position of *Mangrovivirga cuniculi* R1DC9^T^ (MT146883). Only bootstrap values (expressed as percentages of 1000 replications) exceeding 50 % are shown at branching points. *Psychroflexus torquis* ATCC 700755^T^ (GenBank accession no. U85881) was used as an outgroup. Bar, 0.040 substitutions per nucleotide position. Filled circles indicate branches that were also recovered using the neighbour-joining method. (**b**) Maximum-likelihood phylogenetic tree highlighting the position of R1DC9^T^ relative to the other type strains within the order *Cytophagales*, including members of the families *Marivirgaceae*, *Roseivirgaceae*, *Reichenbachiellaceae*, *Fulvivirgaceae*, *Cesiribacteraceae* and *Flammeovirgaceae*. The phylogenetic tree was built using 120 concatenated single-copy genes obtained using GTDB-Tk software [34]. Bootstrap values greater than 50 % based on 1000 replications are indicated at branching nodes. Bar, 0.2 substitutions per nucleotide position.

The comparison of the 16S rRNA gene sequence of R1DC9^T^ with those of related taxa revealed that the closest related species were in the families *Roseivirgaceae* (*Roseivirga echinicomitans* KCTC 12370^T^, *Roseivirga ehrenbergii* KMM 6017^T^, *Roseivirga seohaensis* subsp. *aquiponti* D-25^T^, *Roseivirga spongicola* UST030701-084^T^) and *Marivirgaceae* (*Marivirga sericea* DSM 4125^T^, *Marivirga tractuosa* DSM 4126^T^), which exhibited blast similarities of 89%–89.2 % and 89.5%–89.7 %, respectively. We also noted that a 16S rRNA gene sequence relating to *Flammeovirgaceae* bacterium GY-1 (JX254915) exhibited 97.32 % similarity to our strain; however, as nothing else is known about this species (genome, physiology and chemotaxonomy) we did not include it in the subsequent analyses. The RDP classifier assigned R1DC9^T^ to the family *Flammeovirgaceae* with 99 % confidence, but it designated the strain as an unclassified *Flammeovirgaceae* species with 44 % bootstrap confidence with the genus *Marivirga*. The maximum-likelihood phylogenetic tree based on the 16S rRNA gene sequence placed R1DC9^T^ in a branch separated from the families *Marivirgaceae*, *Cesiribacteraceae*, *Fulvivirgaceae*, *Reichenbachiellaceae* and *Roseivirgaceae* (Fig. 2a). Notably, all these families were previously part of the family *Flammeovirgaceae* [20], explaining the results obtained using the RDP classifier. Similarly, the MLSA phylogenetic tree of 120 concatenated single-copy genes of R1DC9^T^ (obtained from the genome) and related taxa illustrated that our strain formed a separated branch from the aforementioned families (Fig. 2b), supporting that our strain belongs to a new family within the order *Cytophagales*.

*In silico* DDH, AAI and ANIb values were analysed to investigate the genetic relatedness of isolate R1DC9^T^ with 12 fully sequenced genomes representing the families *Cesiribacteraceae*, *Fulvivirgaceae*, *Marivirgaceae*, *Reichenbachiellaceae* and *Roseivirgaceae* (Table 1). The DDH, AAI and ANIb values ranged 17–27.5 %, 55.4–61.4 % and 64–65 %, respectively, compared with the other described species (Table 1). As the standard criterion of DDH is 70 % for species discrimination, and 45–75 % for genus discrimination by AAI and ANIb, respectively [36, 40, 41], our data illustrated that R1DC9^T^ represents a novel genus. Furthermore, to investigate the possibility that R1DC9^T^ forms also a new family, we compared the pairwise AAI values of all sequenced genomes available within the families *Cesiribacteraceae*, *Flammeovirgaceae, Fulvivirgaceae*, *Marivirgaceae*, *Reichenbachiellaceae*, *Roseivirgaceae* and *Thermonemataceae* in the order *Cytophagales*. Results showed that average AAI values among each family range from 65.8%–91.9 % (*i.e.,* all pairwise comparison between genomes of the same family), while average AAI values between families range from 56.7% to 62.6 % (*i.e.*, all pairwise comparisons between genomes of different families; (Fig. S1, available in the online version of this article). As AAI similarities between strain R1DC9^T^ and the other families range from 57.4% to 60.8 %, and a clear branching in the genome-based phylogenetic analysis separated R1DC9^T^ from the aforementioned families (Fig. 2b), the new isolate was classified into the novel genus *Mangrovivirga* as the type species *Mangrovivirga cuniculi* gen. nov., sp. nov. (R1DC9^T^=KCTC 72349^T^=JCM 33609^T^=NCCB 100698^T^) in the new family *Mangrovivirgaceae* of the order *Cytophagales* and the phylum *Bacteroidetes*.

**Table 1.** Average nucleotide identity via blast (ANIb), average amino acid identity (AAI) and *in silico* DNA–DNA hybridization (DDH) matrix of the isolate R1DC9^T^ relative to its closest related type strains The cutoff for species discrimination was ≥70 % for DDH, whereas the cutoffs for genus discrimination was 45% and 75 % for AAI and ANIb, respectively.

Reference genome	Accession no.	ANIb (%)	Aligned nucleotide (%)	DDH (%)	AAI (%)
*Marivirga tractuosa* DSM 4126^T^	GCF_000183425.1	65.9	21.5	18.2	60.2
*Marivirga sericea* DSM 4125^T^	GCF_900177665.1	65.6	19.7	19.9	60.6
*Roseivirga ehrenbergii* KMM 6017^T^	GCF_001593005.1	65.6	18.4	19.9	60.8
*Roseivirga pacifica* CGMCC 1.12402*^T^*	GCF_900111145.1	65.5	16.9	20.1	55.4
*Roseivirga seohaensis* subsp. *aquiponti* D-25^T^	GCF_001267955.1	65.5	18.5	22.1	61.4
*Roseivirga spongicola* UST030701-084^T^	GCF_001592965.1	65.5	17.9	20.1	61.2
*Roseivirga echinicomitans* KMM 6058^T^	GCF_900142205.1	65.4	18.4	22.4	60.1
*Roseivirga misakiensis* SK-8^T^	GCF_001747105.1	65.1	17.4	21.4	60.4
*Reichenbachiella agariperforans* DSM 26134^T^	GCF_900142205.1	65	13.7	21.2	59.6
*Fulvivirga kasyanovii* KMM6220^T^	GCA_009711405.1	65	15.6	20	60.2
*Nafulsella turpanensis* ZLM-10^T^	GCF_000346615.1	64.9	16.2	17	59.8
*Cesiribacter andamanensis* AMV16^T^	GCF_000348925.1	64	12.6	27.5	59.8

## Morphological, physiological and chemotaxonomic characterization

Cells were grown on LB+FSW for 3 days at 37 °C, harvested, washed with distilled water and fixed as described in Supplementary Method S1. Cell morphology was determined using an FEI Teneo scanning electron microscope at the Imaging Core Lab at KAUST. Colony morphology, size and colour were examined on LB+FSW plates incubated for 3 days at 37 °C. Gram staining was performed following the standard protocol [42]. Cell motility was assessed on a tube containing semisolid (0.3 % agar) LB+FSW medium using the hanging drop technique [43, 44]. The effect of temperature (15, 20, 25, 30, 37, 40, 45 and 50 °C) on growth was assessed using LB+FSW medium. Growth of the strain under different salt concentrations (0%–20 % NaCl, increased at intervals of 1 %) was also evaluated using LB medium. The temperature range and NaCl requirement for growth were recorded every 12 h over 4 days of incubation by measuring the optical density at 600 nm using a UV-1600PC spectrophotometer (VWR). Growth at pH 3.5–10 (increased at intervals of 0.5 pH units) was determined using Biolog Phenotype MicroArray PM10 and LB+FSW medium. The Biolog Phenotype Microarray was also used to assess the bacterial growth on different carbon sources (PM1 and PM2 plates) using IF-0a medium supplemented with 4 % NaCl. Growth in the presence of antibiotic compounds was also tested using PM11 and PM12 with LB+FSW medium. Bacterial culture and plate inoculation were performed by using Biolog products and following the manufacturer’s instructions for Gram-stain-negative bacteria; all plates were incubated at 37 °C for 7 days in in the Biolog OmniLog incubator. Additional enzyme activities and biochemical properties were examined. Oxidase activity was determined using oxidase test strips (Sigma-Aldrich). Catalase activity was determined by assessing bubble production in 3 % (v/v) H_2_O_2_ [45]. Indole production was evaluated by adding 500 µl of Kovac’s reagent (Sigma) to bacterial culture grown 3 days in LB+FSW and l-tryptophan. The nitrate reduction reaction was performed using a nitrate reduction kit (Sigma) and nitrate broth prepared with FSW, following the manufacturer’s instructions. Siderophore production was detected qualitatively with blue chrome azurol sulphonate agar plates, mineral phosphate solubilization was determined in Pikovskaya’s liquid medium amended with 0.5 % [Ca_3_(PO_4_)_2_] [46] and indole acetic acid (IAA) production was evaluated in LB+FSW medium supplemented with l-tryptophan using Salkowski’s reagent [47]. Ammonia production was evaluated by growing the bacterial strain in peptone FSW (peptone 10 g l^-1^) and mixing 0.2 ml of the culture supernatant with 1 ml Nessler’s reagent; development of a yellow to brown colour was evaluated as a proxy for ammonia production. Amylase, protease, lipase and cellulase activities were evaluated using LB+FSW medium containing 1.5 % starch, casein, Tween 80 and cellulose, respectively, as substrates. The formation of transparent halos or colour changes in the medium around the colonies indicated positive activities [9]. Analyses of fatty acid, polar lipid and respiratory quinone levels in R1DC9^T^ cells cultivated in LB+FSW medium were performed by the Identification Service and Dr. Brian Tindall at the DSMZ (German Collection of Microorganisms and Cell Cultures GmbH, Braunschweig, Germany).

**Fig. 3.**
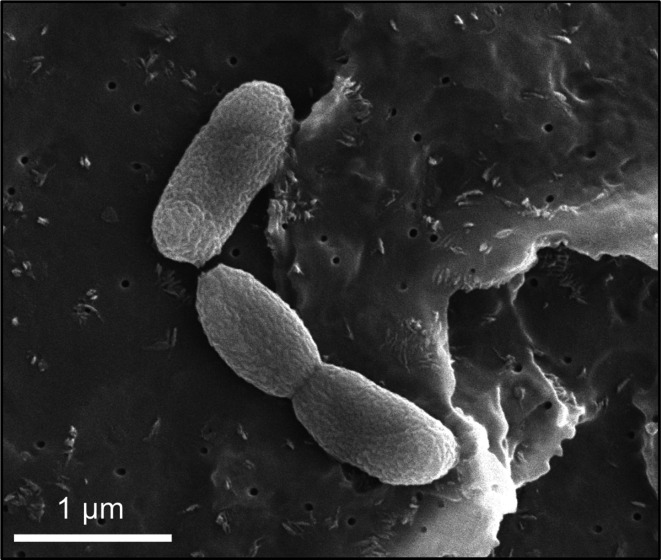
Scanning electron microscopy image of *Mangrovivirga cuniculi* R1DC9^T^ (bar, 1 µm).

Cells of strain R1DC9^T^ were Gram-stain-negative, non-spore-forming, strictly aerobic and rod-shaped (0.3–0.5 µm wide, 1–1.2 µm long; Fig. 3). When R1DC9^T^ was cultivated on LB+FSW for 48 h at 37 °C, its colonies were circular with regular edges, smooth, shiny, orange and 1–2 mm in diameter. R1DC9^T^ grew between 20 and 40 °C (optimum, 37 °C; Fig. S2a). The permissive pH range for growth was pH 6–10 (optimum, pH 8). The doubling time of the bacterium under optimal growth conditions was 6.5 h. The strain grew in the presence of 3%–11% NaCl (optimum, 7%–9 %) at 37 °C (Fig. S2b), indicating that the strain was halophilic [48]. This result revealed an adaptation to/dependence on the saline conditions of the mangrove sediments of the Red Sea, in which dilution with freshwater never occurs because of the extremely limited freshwater input (no rivers and extremely rare rainfall), and the sediments are cyclically water-logged by seawater during tides [6, 49]. The carbon sources used by R1DC9^T^ for growth included pectin, 2-deoxy-d-ribose, d-ribose, 5-keto-d-gluconic acid, l-ornithine and dihydroxyacetone. The strain exhibited weak growth in the presence of thymidine, uridine and adenosine as the sole carbon source. The strain was positive for cytochrome oxidase, lipase activity and nitrate reduction, and it was negative for catalase, cellulase, amylase, protease and indole production. In addition, among the plant growth promoting (PGP) traits tested, R1DC9^T^ was negative for siderophore production and phosphate solubilization, whereas it was positive for auxin (IAA) and ammonia production. While indole and IAA are catalysed from tryptophane, two different enzymes classes are responsible for their formation (tryptophanase and tryptophan *N*-monooxygenase for indole and IAA, respectively [50, 51]), thus explaining why R1DC9^T^ cannot produce indole but can produce IAA. The morphological, physiological and biochemical characteristics of R1DC9^T^ and its closest relatives are summarized in (Table 2). We chose these closest relatives based on their 16S rRNA gene sequence similarities to R1DC9^T^. Regarding antibiotics, R1DC9^T^ was susceptible (*i.e.* bacterial growth was inhibited at all four antibiotic concentrations present in Biolog Phenotype Microarray plates) to rifampicin, spiramycin, penicillin G, nafcillin, lincomycin, cloxacillin, oxacillin, vancomycin, novobiocin and erythromycin, whereas strain R1DC9^T^ was resistant (*i.e*., bacterial growth was observed at all four antibiotic concentrations present in Biolog plates) to spectinomycin, amikacin, kanamycin, lomefloxacin, gentamicin, neomycin, amoxicillin, bleomycin, colistin, minocycline, capreomycin, demeclocycline, tetracycline, sulfathiazole, sulfamethazine, sulfadiazine, sulfamethoxazole, paromomycin, sisomicin, tobramycin, cefazolin, enoxacin and ceftriaxone (Table S1). R1DC9^T^ displayed resistance to low concentrations (*i.e.*, first and second wells of each antibiotic in Biolog Phenotype Microarray plates) of chloramphenicol, chlortetracycline, carbenicillin, penimepicycline, polymyxin B, potassium tellurite, cephalothin and ofloxacin. It is important to note that antibiotic resistance is strain-specific; thus, these results are only attributable to the isolated strain.

**Table 2.** General features and genomic and phenotypic characteristics of R1DC9^T^ and its closest related members of the order *Cytophagales* Strains: 1, R1DC9^T^ (data from this study); 2, *Marivirga tractuosa* DSM 4126^T^ [17]; 3, *Roseivirga ehrenbergii* SW-152^T^ [18]; 4, *Roseivirga misakiensis* SK-8^T^ [62]; 5, *Fulvivirga kasyanovii* KMM 6220^T^ [63]. Characteristics are scored as (+) positive, (−) negative or no data available (na).

Characteristic	1	2	3	4	5
General features:					
Habitat	Mangrove sediment	Beach sand	Seawater	Coastal water	Seawater
Morphology	Rod	Long-rod	Rod	Rod	Rod
Pathogenicity	−	−	−	−	−
Growth at/with:					
Temperature (optimum)	20–40 (37)	10–40 (28–32)	4–40 (30)	10–30 (20–25)	14–44 (35–37)
NaCl (optimum)	3–11 (7–9)	0.5–10 (4–7)	2–9 (2–3)	1–5	0–10 (2–3)
pH (optimum)	6–10 (8)	na	5.5–9 (7–8)	6–10 (7–9)	na
Genome features:					
Genome size (bp)	4 661 901	4 516 490	4 160 330	4 452 385	7 174 826
DNA G+C content (mol%)	36.1	35.5	40.3	39.1	59.9
Predominant menaquinone	MK-7	MK-7	MK-7	MK-7	MK-7
Utilization of:					
d-Glucose	−	+	−	+	−
l-Arabinose	−	+	−	na	na
d-Mannose	−	+	−	−	−
Arbutin	−	+	na	+	na
l-Ornithine	+	−	na	na	−
d-Ribose	+	na	−	na	na
Maltose	−	+	−	−	+
Mannitol	−	+	−	+	−
Sucrose	−	+	−	−	−
Sorbitol	−	+	−	na	−
Enzyme activities:					
Oxidase	+	+	+	+	+
Catalase	−	+	+	+	+
Cellulase	−	−	+	+	−
Amylase	−	+	+	+	−
Lipase	+	−	−	−	−
Nitrate reduction	+	−	−	−	−
Indole production	−	−	−	−	−

The major fatty acids (>5 %) of R1DC9^T^ included the saturated branched chain fatty acids iso-C_15 : 0_ (57.1 %), iso-C_17 : 0_ 3-OH (12.8 %) and iso-C_17 : 0_ (5.1 %), whereas the monounsaturated fatty acid C_16 : 1_ *ω*5*c* comprised 5.8 % of the total fatty acid content. The cellular fatty acid composition may change depending on the medium where cells have been grown, so the comparison of R1DC9^T^ fatty acids with the published fatty acids of strains from the related families should be taken with caution, because fatty acids of strain R1DC9^T^ were obtained from cells cultivated on LB+FSW, while most of the other compared strains were grown in marine broth (Table 3). R1DC9^T^ differed from related families in the order *Cytophagales* based on its significantly higher proportion of iso-C_15 : 0_. The presence of C_16 : 1_ *ω*5*c* was also detected, whereas unsaturated chain fatty acids were poorly represented. The major respiratory quinone was MK7, which is the predominant quinone in the order *Cytophagales*. The polar lipid profile of R1DC9^T^ included one phosphatidylethanolamine, two unknown phospholipids and two unknown lipids. Meanwhile, the absence of two aminolipids, one glycolipid and two unidentified polar lipids differentiated R1DC9^T^ from the related species of the genera *Marivirga* and *Roseivirga*. Taken together, the chemotaxonomic results confirmed that R1DC9^T^ belongs to the order *Cytophagales* but differs from the current families belonging to said order.

**Table 3.** Cellular fatty acid composition (%) of R1DC9^T^ (grown in LB+FSW) and closely related members (generally, grown in marine broth; growth medium specifications in the reference articles in the note) of *Cytophagales* Strains: 1, R1DC9^T^ (data from this study); 2, *Marivirga tractuosa* DSM 4126^T^ [17]; 3, *Roseivirga ehrenbergii* SW-152^T^ [64]; 4, *Roseivirga misakiensis* SK-8^T^ [62]; 5, *Fulvivirga kasyanovii* KMM 6220^T^ [63]. nd, Not detected. Bold indicates the prevalent fatty acid components in strain R1DC9^T^ (*i.e.*, > 5 %). Fatty acids accounting for less than 1 % in all the strains are not reported; tr, trace (<1 %).

Fatty acid	1	2	3	4	5
Saturated straight chain:					
C_16 : 0_	2.8	–	–	–	–
C_15 : 0_	tr	4.4	–	–	–
Saturated branched chain:					
iso-C_13 : 0_	tr	1.2	4.3	–	–
iso-C_15 : 0_	**57.1**	36.8	26.4	18.1	31.2
anteiso-C_15 : 0_	tr	–	4.3	2.9	–
iso-C_16 : 0_	tr	3.7	tr	–	–
iso-C_17 : 0_	**5.1**	1.2	tr	–	–
Unsaturated branched chain:					
iso-G-C_15 : 1_	1.5	23	27.0	47.6	5.1
iso-G-C_16 : 1_	–	1.4	tr	1.8	–
C_16 : 1_ *ω*5*c*	**5.8**	–	–	–	–
anteiso-C_15 : 1_	–	–	0.8	–	1.71
Hydroxylated:					
iso-C_15 : 0_ 3-OH	3.2	–	5.7	6.2	7.0
C_16 : 0_ 3-OH	3.4	2.8	1.9	6.2	–
iso-C_16 : 0_ 3-OH	tr	2.8	5.7	4.7	–
C_17 : 0_ 2-OH	–	–	1.4	–	–
iso-C_17 : 0_ 3-OH	**12.8**	12.2	11.1	9.8	23.7
Summed feature 3*	tr	tr	2.4	–	24.7
Summed feature 4*	1.8	–	–	–	–

*Summed features are groups of two or three fatty acids that could not be separated via GLC using the midi system. Summed feature 3 comprises iso-C_15 : 0_ 2-OH and/or C_16 : 1_ *ω*7*c* and/or C_16 : 1_ *ω*6*c* and/or and/or iso-C1_5 : 0_ 2-OH. Summed feature 4 comprises iso-C_17 : 1_ and/or anteiso-C_17 : 1_ B.

## Genome features

The genome of R1DC9^T^ is 4 661 901 bp long and its G+C content is 36.1 mol% (Fig. S3a). Among the 4316 annotated genes, 4247 are protein-coding sequences (CDS) and 12 are rRNA-coding genes, including four identical 16S rRNA genes (1544 bp) and 43 tRNA. In total, 54 % of the protein-coding genes were assigned putative functions, whereas the remaining genes were annotated as hypothetical proteins. As noted for other *Bacteroidetes* species, the R1DC9^T^ genome revealed the presence of polysaccharide-utilization loci [52]. We detected the presence of genes involved in osmoprotectant biosynthesis, such as *proC* (proline biosynthesis, DCC35_RS09745), *lysC*/*asd* (ectoine biosynthesis, DCC35_RS01520) and *glgA*/*glgB* (trehalose biosynthesis, DCC35_RS02325, DCC35_RS11445). These compounds are used by halophilic bacteria to counteract the effects of salinity and related osmotic stress induced by high concentrations of salt ions in the ‘salt-out’ strategy [53, 54]. In addition, we identified the transporter genes *opuBA*, *opuBB* and *opuBC* (DCC35_RS10255, DCC35_RS10260) involved in the import of osmoprotectants, such as glycine betaine/proline, inside the cells. To confirm the use of the ‘salt-out’ strategy by R1DC9^T^ (*i.e.,* the use of osmoprotectants to counteract the effects of salinity), we inferred the isoelectric point (*p*I) of the proteome using the ExPASy server [55]. R1DC9^T^ had a sub-acidic *p*I, similar to that of the halophilic strain *Desulfohalobium retbaense* DSM 5692^T^ known to use the ‘salt-out’ strategy [56] and slightly lower than those of the closest known species (*R. ehrenbergii* SW-152^T^, *R. misakiensis* SK-8^T^, *M. tractuosa* DSM 4126^T^; Fig. S3b), whereas the halophilic bacterium *Salinibacter ruber* M31^T^ (Fig. S3b) which adopts a ‘salt-in’ strategy in which K^+^ ions are accumulated in the cytoplasm and adaptation of the cellular machinery to a charged cytoplasm and acid proteome is necessary, had an acidic *p*I [57]. These results confirmed the capacity of our strain to quickly adapt to changing salinity occurring in mangroves as the production and/or importation of osmoprotectants by the cell is a high-turnover mechanism.

Additionally, the genome of R1DC9^T^ contains several genes encoding proteins involved in the production of carotenoids (*crtB*, *lcyB*, *crtI*, *crtO*, *crtZ*, *miaA*, *ispB*, DCC35_01745, DCC35_05035, DCC35_01740, DCC35_05045, DCC35_RS13000, DCC35_RS01730, DCC35_RS08495, DCC35_RS06995), indicating the ability to synthesize pigments, such as beta-carotene, astaxanthin and lycopene, which counteract photooxidative stress under unfavourable conditions [58] by quenching singlet oxygen and lipid peroxidation or scavenging hydroxyl radicals [59, 60]. Although some of the genes related to gliding motility were predicted in the genome of this bacterium (*gldA*, *gldC*, *gldD*, *gldE*, *gldF*, *glcG*, *gldJ*, *gldL*, *gldM*, *gldN*, DCC35_RS07255, DCC35_RS14410, DCC35_RS02560, DCC35_RS02550, DCC35_RS07260, DCC35_RS07265, DCC35_RS02080, DCC35_RS02985, DCC35_RS02990, DCC35_RS02995), it was non-motile in an *in vitro* test in semisolid medium. However, we noted the absence of some of the genes of the cluster (*gldB*, *gldH*, *gldK*, *gldI*), which could explain the absence of gliding motility [61]. As this strain was found in bioturbated sediments, the sole strict adhesion to surfaces may allow it to thrive in an environment in which nutrients are constantly renewed by the tide cycles and crab bioturbation [6]. Regarding PGP genes, it should be noted that none of the known proteins involved in IAA production [50] could be found with a high enough identity (>60 %) to confirm their presence in our genome; the closest match possessed only a 40 % identity with indole-3-acetaldehyde dehydrogenase, which is part of the indole-3-pyruvate pathway [50]. This is probably due to the fact that our strain is part of a novel family obtained from a poorly studied environment and with a limited number of closest-related genomes available. We also noted that R1DC9^T^ possesses genes involved in the ammonia production via the dissimilatory nitrate reduction pathway (*nrfA*, *nrfH*, DCC35_16515, DCC35_16510).

The low level of 16S rRNA gene sequence similarity, the independent phylogenetic position, the relatively low AAI, ANIb and DDH values, and the differences in numerous phenotypic properties, cellular fatty acid composition, polar lipid profiles and DNA G+C content between R1DC9^T^ and its closest phylogenetic described species (*M. tractuosa* DSM 4126^T^, *R. ehrenbergii* SW-152^T^, *R. misakiensis* SK-8^T^ and *F. kasyanovii* KMM 6220^T^) indicated that the strain diverged from such taxa. Therefore, we suggest that R1DC9^T^ represents a novel species in a novel genus within the new family *Mangrovivirgaceae* of the order *Cytophagales*, for which the name *Mangrovivirga cuniculi* gen. nov., sp. nov. is proposed.

## Description of *Mangrovivirga* gen. nov.

*Mangrovivirga* (Man.gro.vi.vir′ga. N.L. neut. n. *mangrovum* a mangrove; L. fem. n. *virga* rod; N.L. fem. n. *Mangrovivirga* for a mangrove rod, referring to the isolation of a rod-shaped bacterium from the mangrove environment).

Cells of the species are strictly aerobic, Gram-stain-negative, long-rod-shaped, moderately halophilic, non-spore-forming, non-motile, catalase-positive and oxidase-negative. The major respiratory quinone is MK7 and the major polar lipids are phosphatidylethanolamine, two unknown phospholipids and two unknown lipids. The major cellular fatty acids (>5 %) are iso-C_15 : 0_, C_16 : 1_ *ω*5*c*, iso-C_17 : 0_ and iso-C_17 : 0_ 3-OH. The G+C content of the genomic DNA is 63.1 mol%. The type species is *Mangrovivirga cuniculi*.

## Description of *Mangrovivirga cuniculi* sp. nov.

*Mangrovivirga cuniculi* (cu.ni.cu′li. L. gen. n. *cuniculi* of a burrow, named because the type species was isolated from a crab burrow).

The cell morphology and chemotaxonomic characteristics are given in the genus description. Cells are 0.3–0.5 µm wide and 1–1.2 µm long. Its colonies are circular with a diameter of 1–2 mm and feature regular edges, a smooth and shiny surface, and an orange colour caused by the production of orange carotenoids. The permissive conditions for growth are a temperature of 20–40 °C (optimum, 37 °C), pH 6–10 (optimum, pH 8) and 3%–11 % NaCl for salinity (optimum, 7%–9 % NaCl). The carbon sources used for growth are pectin, 2-deoxy-d-ribose, d-ribose, 5-keto-d-gluconic acid, l-ornithine, dihydroxyacetone, thymidine, uridine and adenosine. The genome harbours genes responsible for protection against oxidative, osmotic and salinity stresses, and includes genes encoding proteins that produce osmoprotectants and carotenoids. The cells are negative for amylase, protease, lipase, cellulase, indole, siderophore production and phosphate solubilization, and are positive for auxin (IAA) and ammonia production. The strain is unable to reduce nitrate to nitrite.

The type strain, R1DC9^T^ (=KCTC 72349^T^=JCM 33609^T^=NCCB 100698^T^), was isolated from bioturbated mangrove sediment at the Ibn Sina Field Research Station and Nature Conservation Area in KAUST, Saudi Arabia. The genomic DNA G+C content and genome size of the type strain are 63.1 mol% and 4 661 901 bp, respectively.

## Description of *Mangrovivirgaceae* fam. nov.

*Mangrovivirgaceae* (Man.gro.vi.vir′ga’ce.ae, N.L. fem. n. *Mangrovivirga* type genus of the family; -aceae, ending to denote a family; N.L. fem. pl. n. *Mangrovivirgaceae*, the family of the genus *Mangrovivirga*).

On the basis of 16S rRNA gene and genome sequence analyses, the species is a member of the order *Cytophagales* in the phylum *Bacteroidetes*. The description is the same as that for the genus *Mangrovivirga*, which is the type and currently sole genus of the family.

## Supplementary Data

Supplementary material 1
