# Supplementary material for: Mangrovivirga cuniculi gen. nov., sp. nov., a moderately halophilic bacterium isolated from bioturbated Red Sea mangrove sediment, and proposal of the novel family Mangrovivirgaceae fam. nov
Source: Int J Syst Evol Microbiol. 2021 Jul 2;71(7):004866. doi: 10.1099/ijsem.0.004866 (PMC8489838; doi:10.1099/ijsem.0.004866)
Supplement: Supplementary material 1 [file ijsem-71-4866-s001.pdf]

Supplementary Material

Supplementary Method S1. Scanning Electron Microscopy analysis of R1DC9^T. Cells of strain R1DC9^T were cultivated on FSW+LB liquid medium; 1 ml of liquid culture grown for 48 hours was collected in 2 ml tube and centrifuged at 2000 rpm for 5 min. Bacterial cells were fixed with 2.5 % glutaraldehyde in 0.1 M cacodylate buffer pH 7.4, and stored at 4°C of overnight. Cells were washed in 0.1 M cacodylate buffer three times for 15 min, and placed on cover slip 18 × 18 coated with L-lysin 2 mg/ml, and kept to dry overnight. Cover slip with bacterial cells was post-fixed in osmium 1% in 0.1 cacodylate buffer for 1 hour in dark, washed in distilled water three times for 15 min, dehydrated treating the cover slip with an ethanol gradient (30, 50, 70, 90, 100 %) for 15 min each, and subsequently dried by critical point (Leica EM CPD300, Leica). Bacterial cells were coated with Pt/Pd using a K575X sputter coater (Quorum) and their morphology determined by using Teneo scanning electron microscope (SEM) at the Imaging Core Lab at King Abdullah University of Science and Technology.

Supplementary Figure S1. Comparison of the average amino acid identity (AAI) between sequenced members from different families. The percentage of AAI is indicated in the figure. In red, AAI comparison between genomes of the same family. In blue, AAI comparison between members of different families. In bold, AAI comparison between *Mangvrovivigaceae* and other families. The number in parenthesis indicate the number of sequenced genomes belonging to the same family.

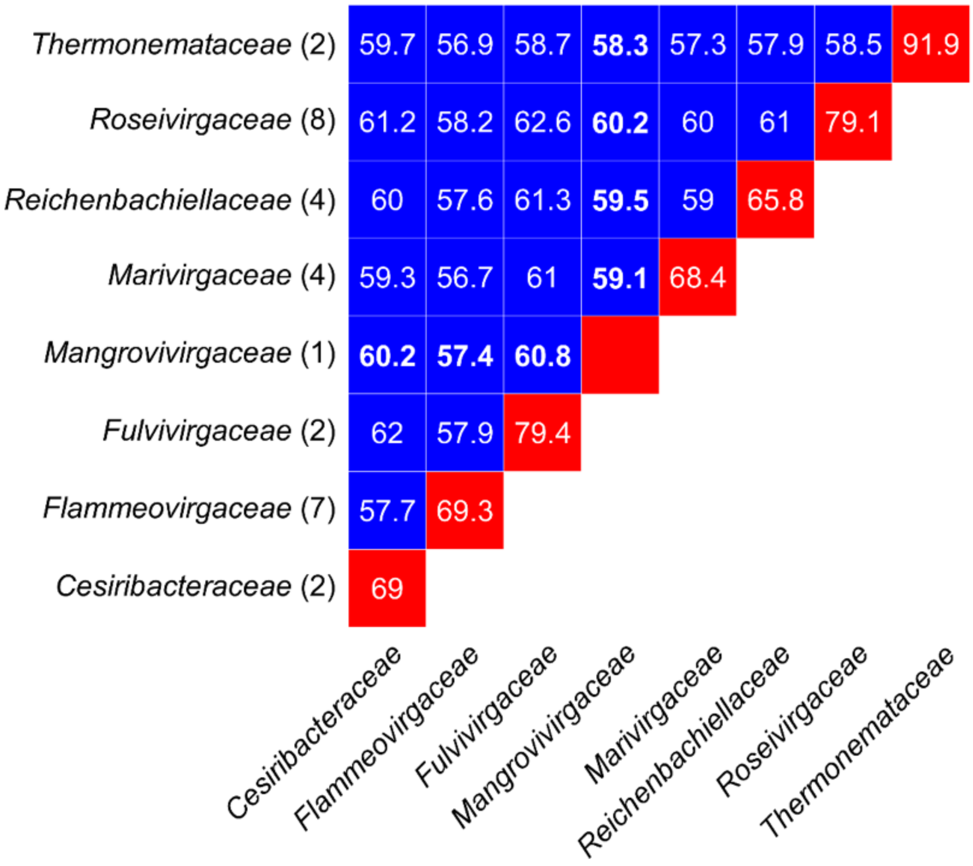

Supplementary Figure S2. (**a** and **b**) Heat map representing the growth of R1DC9^T at different temperatures (range, 15°C–50°C in LB+FSW) and salinity concentrations (NaCl, 0%–15% NaCl at 37°C) in LB + 10% FSW, respectively. Values are reported as optical density at 600 nm (OD_{600}) and expressed as the mean of three replicates. LB, lysogeny broth; FSW, filtered seawater.

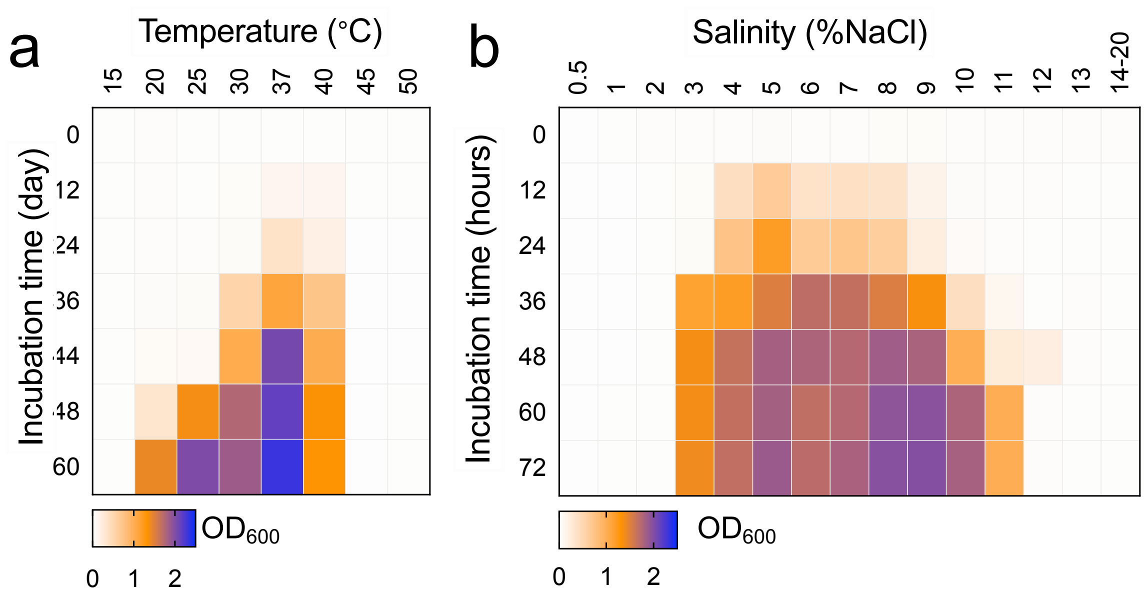

Supplementary Figure S3. (**a**) Graphical circular map of the chromosome and table resuming the genome features of *M. cuniculi* strain R1DC9^T. From outside to the centre: genes on forward strand, genes on reverse strand, RNA genes (tRNAs purple, rRNAs blue), G+C content, G+C skew. (**b**) Isoelectric point of the proteome of *D. retbaense* DSM 5692^T [1], *R. seohaensis* SW-152^T [2], *R. misakiensis* SK-8^T [3], *M. tractuosa* DSM 4126^T [4], *M. cuniculi* R1DC9^T and, *S. ruber* M31^T [5].

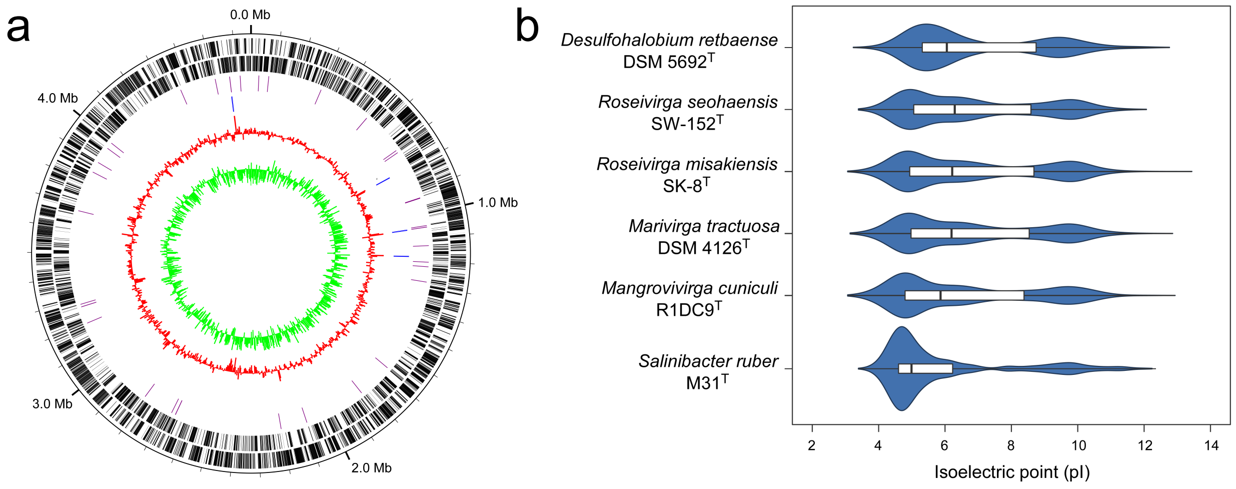

Supplementary Table S1. Antibiotic sensitivity test of R1DC9^T on Biolog PM11 and PM12 microplates. Strain sensitivity to antibiotics is scored; Biolog PM plates contain 4 different concentrations for each antibiotic (from lower to highest; information regarding the quantities is not available), and we classified the sensitivity/resistance capacity of our strain based on its ability to growth at the different concentration. For instance, antibiotic sensitive (S), *i.e.*, the bacterial growth is inhibited at all the four antibiotic concentration available in the PM plates; antibiotic resistance (R), *i.e.*, the bacterial grow at all the concentration of the antibiotic; intermediate resistance (IR), *i.e.*, bacteria can growth only in presence of low concentrations of the antibiotic (1 and 2 wells of antibiotic set).

**Antibiotic class (Biolog, PM11)**		**Sensitivity**	**Antibiotic class (Biolog, PM12)**		**Sensitivity**
**Aminoglycosides**	Amikacin	R	**Glycopeptides**	Bleomycin	R
	Neomycin	R		Colistin	R
	Gentamicin	R		Capreomycin	R
	Kanamycin	R		Polymyxin B	IR
	Paromomycin	R		Vancomycin	S
	Sisomicin	R	**Tetracyclines**	Chlortetracycline	IR
	Novobiocin	S		Minocycline	R
	Tobramycin	R		Demeclocycline	R
	Spectinomycin	R		Tetracycline	R
**β- lactams**	Amoxicillin	R		Penimepicycline	IR
	Cloxacillin	S	**Amphenicols**	Chloramphenicol	IR
	Nafcillin	S	**Macrolides**	Erythromycin	S
	Cefazolin	R		Spiramycin	S
	Ceftriaxone	R	**Sulfonamides**	Sulfamethoxazole	R
	Cephalothin	IR		Sulfathiazole	R
	Penicillin G	S		Sulfadiazine	R
	Carbenicillin	IR		Sulfamethazine	R
	Oxacillin	S	**Rifamycins**	Rifampicin	S
**Lincosamides**	Lincomycin	S			
**Synthetic antibiotics**	Lomefloxacin	R			
	Enoxacin	R			
	Nalidixic acid	IR			
	Ofloxacin	IR			

References

1. **Spring S, Nolan M, Lapidus A, del Rio TG, Copeland A, *et al.*** Complete genome sequence of Desulfohalobium retbaense type strain (HR 100T). *Stand Genomic Sci* 2010;2:38–48.
2. **Selvaratnam C, Thevarajoo S, Goh KM, Chan KG, Chong CS**. Proposal to reclassify roseivirga ehrenbergii (Nedashkovskaya et al., 2008) as roseivirga seohaensis comb. nov., description of roseivirga seohaensis subsp. aquiponti subsp. nov. and emendation of the genus Roseivirga. *Int J Syst Evol Microbiol* 2016;66:5537–5543.
3. **Wong SK, Park S, Lee JS, Lee KC, Chiura HX, *et al.*** Fabibacter misakiensis sp. Nov., a marine bacterium isolated from coastal surface water. *Int J Syst Evol Microbiol* 2015;65:3276–3280.
4. **Nedashkovskaya OI, Vancanneyt M, Kim SB, Bae KS**. Reclassification of Flexibacter tractuosus (Lewin 1969) Leadbetter 1974 and 'Microscilla sericea' Lewin 1969 in the genus Marivirga gen. nov. as Marivirga tractuosa comb. nov. and Marivirga sericea nom. rev., comb. nov. *Int J Syst Evol Microbiol* 2010;60:1858–1863.
5. **Antón J, Oren A, Benlloch S, Rodríguez-Valera F, Amann R, *et al.*** Salinibacter ruber gen. nov., sp. nov., a novel, extremely halophilic member of the Bacteria from saltern crystallizer ponds. *Int J Syst Evol Microbiol* 2002;52:485–491.
6. **Nedashkovskaya OI, Kim SB, Shin DS, Beleneva IA, Mikhailov V V.** Fulvivirga kasyanovii gen. nov., sp. nov., a novel member of the phylum Bacteroidetes isolated from seawater in a mussel farm. *Int J Syst Evol Microbiol* 2007;57:1046–1049.
